# The effect of a micro-visual intervention on the accelerated recovery of patients with kinesiophobia after total knee replacement during neo-coronary pneumonia

**DOI:** 10.1097/MD.0000000000024141

**Published:** 2021-02-12

**Authors:** Guanzhen Lu, Tingting Wu, Qin Tan, Zhe Wu, Lingmei Shi, Yan Zhong

**Affiliations:** a Department of Orthopaedics, Huzhou Central Hospital, Zhejiang Province AND Central Hospital affiliated to Huzhou University; b Department of Cardiology, Zhejiang Province, First Affiliated Hospital of Wenzhou Medical University.

**Keywords:** accelerated rehabilitation, akinesiophobia, knee arthroplasty, micro-video

## Abstract

**Background:**

The global neo-coronary pneumonia epidemic has increased the workload of healthcare institutions in various countries and directly affected the physical and psychological recovery of the vast majority of patients requiring hospitalization in China. We anticipate that post-total knee arthroplasty kinesiophobia may have an impact on patients’ postoperative pain scores, knee function, and ability to care for themselves in daily life. The purpose of this study is to conduct a micro-video intervention via WeChat to verify the impact of this method on the rapid recovery of patients with kinesiophobia after total knee arthroplasty during neo-coronary pneumonia.

**Methods:**

Using convenience sampling method, 78 patients with kinesiophobia after artificial total knee arthroplasty who met the exclusion criteria were selected and randomly grouped, with the control group receiving routine off-line instruction and the intervention group receiving micro-video intervention, and the changes in the relevant indexes of the two groups of patients at different time points on postoperative day 1, 3 and 7 were recorded and analyzed.

**Results:**

There were no statistical differences in the scores of kinesiophobia, pain, knee flexion mobility (ROM) and ability to take care of daily life between the two groups on the first postoperative day (*P* > .05). On postoperative day 3 and 7, there were statistical differences in Tampa Scale for kinesiophobia, pain, activities of daily living scale score and ROM between the two groups (*P* < .01), and the first time of getting out of bed between the two groups (*P* < .05), and by repeated-measures ANOVA, there were statistically significant time points, groups and interaction effects of the outcome indicators between the 2 groups (*P* < .01), indicating that the intervention group reconstructed the patients’ postoperative kinesiophobiaand hyperactivity. The level of pain awareness facilitates the patient's acquisition of the correct functional exercises to make them change their misbehavior.

**Conclusions:**

WeChat micro-video can reduce the fear of movement score and pain score in patients with kinesiophobia after unilateral total knee arthroplasty, shorten the first time out of bed, and improve their joint mobility and daily living ability.

**Ethics:**

This study has passed the ethical review of the hospital where it was conducted and has been filed, Ethics Approval Number: 20181203-01.

## 1 Introduction

The knee joint is the largest and most complex complex joint in the human body and carries 4 to 6 times the longitudinal gravitational force of body weight.^[1]^ The knee joint is 1 of the lower limb joints that assists people in performing important activities such as squatting and knee bending, mainly helping to maintain postures related to human movement and performing essential activities. With the aging of the world population, the number of patients who undergo total knee arthroplasty (TKA) every year is increasing rapidly, and the majority of them are middle-aged and elderly.^[2]^ Culliford found that the number of TKA patients in the United Kingdom has increased rapidly in the last decade, and it is predicted that by 2030, the number of TKA patients will reach 1.22 million, which is twice as many as in 2010.^[3]^ Based on the disadvantages of the procedure, such as high trauma and bleeding, patients tend to experience more severe pain after surgery, with up to 60% of patients in early severe pain.^[4]^

Because of the special physiological structure and position of the knee joint, most of the patients will have severe pain after TKA. Faced with the stimulation of pain caused by multiple factors, some patients may think that activities or functional exercises will aggravate the postoperative pain or cause re-injury to the knee joint.^[5]^ The incidence of kinesiophobia is 52.8%, which affects the patient's recovery after surgery.^[6]^ kinesiophobia is an irrational psychological state in which the patient, when faced with painful stimuli, has the misconception that activity or rehabilitation exercises will lead to re-injury or aggravate pain, and is therefore unwilling to actively engage in rehabilitation exercises.^[7]^ Kinesiophobia is an important factor in the prognosis of total knee arthroplasty, and in severe cases, it can affect the recovery of joint function and even lead to joint disuse and disability.

WeChat can be used to educate patients about the disease and can be communicated in various forms, such as voice, text and even video, which is more suitable for rehabilitation guidance during neocoronary pneumonia.^[8,9]^ This study aims to help patients with kinesiophobia after unilateral total knee arthroplasty to carry out early rehabilitation exercises through short micro-videos in order to reduce their kinesiophobiaa and pain level, to help them master the correct functional exercises, to help improve knee function, and to speed up the rehabilitation.

## 2 Research methodology

### 2.1 Ethics

This study has passed the ethical review of the hospital in which it was conducting (Ethical Approval No. 20181203-01), and the project was established and supported by the Provincial Medical and Health Science and Technology Program (2020KY936) during the research process. Ethics-related contents were strictly observed in the study.

### 2.2 Research design

This study is a prospective, randomized, single-blind (subject), controlled clinical study. The purpose and procedure of the study will be informed and explained by the investigator and the nurse in charge before the start of the intervention, and it informed consent for the study will be signed after obtaining the patient's approval and understanding. This study does not additionally increase the risk of post-operative rehabilitation, and the relevant rehabilitation exercises in the study will be carried out in the company of a rehabilitation therapist, if any adverse reaction occurs, it will be immediately stopped and contacted and dealt with by the rehabilitation therapist and physician in the current ward, the relevant compensation measures and insurance by the hospital rules and procedures.

### 2.3 Subjects of study

Patients who were admitted to our hospital for unilateral total knee arthroplasty from January 2020 to June 2020 during the period of neo-coronary pneumonia resistance, were selected by convenience sampling method and scored by Tampa Scale for kinesiophobia (TSK) within 24 hour after waking up from anesthesia according to the inclusion-exclusion criteria. One patient in the intervention group and 1 patient in the control group withdrew from the study, with a loss of follow-up rate of 2.5%, and the general information of patients is detailed in Table 1.

**Table 1:** Comparison of basic information of 2 groups of patients.

Characteristics	Levels	Intervention	Control	*t*-test	Significance
Age		69.28 ± 6.85	68.79 ± 5.80	0.339	0.736
BMI		24.51 ± 3.33	25.64 ± 3.44	−1.471	0.145
Years of pain		6.92 ± 6.37	4.93 ± 4.20	1.635	0.106
		n (%)	n (%)	X^2^	significance
Genders					
	Man	11 (14.1)	9 (11.5)	0.269	0.604
	Women	28 (35.9)	30 (38.5)		
Marital status)					
	Married	38 (48.7)	37 (47.4)	0.347	0.556
	Unmarried	1 (1.3)	2 (2.6)		
Education level					
	Primary schools	13 (16.7)	17 (21.8)	2.314	0.540
	Junior high school	18 (23.1)	14 (17.9)		
	High school	5 (6.4)	7 (9.0)		
	College and above	3 (3.8)	1 (1.3)		
Career					
	Peasants	26 (33.3)	30 (38.5)	1.347	0.518
	Employees	3 (3.8)	3 (3.8)		
	Other	10 (12.8)	6 (7.7)		
Type of health insurance					
	New rural cooperative medical insurance	14 (17.9_	10 (12.8)	1.864	0.394
	Urban health insurance	16 (20.5)	22 (28.2)		
	All expenses paid	9 (11.5)	7 (9.0)		
Surgical Location					
	Left knee	21 (26.9)	20 (25.6)	0.051	0.821
	Right knee	18 (23.1)	19 (24.4)		

Inclusion criteria:

(1) patients who met the American Rheumatism Association (ARA) diagnostic criteria for knee osteoarthritis and underwent total knee arthroplasty,^[10]^
(2) patients with TSK score >37 within 24 hours after surgery,^[11]^
(3) patients who underwent unilateral total knee arthroplasty for the first time,
(4) patients who voluntarily participated in this study and signed an informed consent form.

Exclusion Criteria:

(1) patients with lower extremity thrombosis or previous history of embolism and need to restrict lower extremity activities,
(2) patients with other lower extremity joint pathologies such as ankle and hip,
(3) patients with illiteracy, confusion, mental or communication disorders,
(4) patients who have participated in other similar studies.

### 2.4 Research tools

(1) General information: including the patient's name, bed number, age, BMI, surgical location, education level, and marital status.
(2) The TSK: This scale was proposed by Kori^[7]^ in 1990 and applied to chronic pain patients, and was later developed and validated by Hu in China.
(3) Besides, the Pain Score, Joint Mobility, and Daily Living Skills Self-Care Scale was also included.

### 2.5 Statistical methods

SPASS21.0 software was used to statistically and analytically analyze patients’ general information and related indicators by using Excel software to enter the database and adopting a 2-checking method. Patients’ measurement data were described by mean and standard deviation, and statistical analysis was performed by two independent samples *t*-test and non-parametric rank-sum test; patients’ counting data were described by frequency and percentage, and statistical analysis was performed by karyotype test; patients’ monitoring data were analyzed by Repeated Measures ANOVA (Repeated Measures ANOVA). Trends in the indicator over time, where *P* < .05 is different.

### 2.6 Research methodology

#### 2.6.1 The control group approach

The control group gave direct and regular verbal and face-to-face instructions to the patients, as follows: when the patients woke up from anesthesia, they were told to perform ankle pump exercise and quadriceps muscle contraction exercise, etc. They were instructed to increase or decrease the amount of exercise according to their state and degree of muscle fatigue; 1 day after the operation, they were instructed to bend their knee joints and straight leg raising exercise, etc. After that, the patients could adjust appropriately according to their rehabilitation situation. During this period, the patient's condition is closely observed and the vital signs are recorded regularly.

#### 2.6.2 Intervention group methods

The intervention group used WeChat for online mobile phone micro-video broadcasting. The researcher could provide simultaneous cognitive belief education and exercise behavior essentials instruction next to the patient during the micro-video broadcast. Micro-video viewing is primarily the responsibility of the patient's charge nurse, and is scheduled for afternoon rounds by the charge nurse. The frequency is once a day, and the video can be given to the patient free of charge before discharge if the patient needs it. The micro-videos were disseminated in 3 parts: actionable exercise goals, detailed demonstration of movement and breakdown, and peer and health care support education. Patients were monitored on the first, third and seventh postoperative days to evaluate the dissemination effect, including the Tampa score for agoraphobia, knee mobility (maximum flexion mobility), pain classification, and Bartholomew's scale of self-care ability in daily life (including getting out of bed, eating and modification).

Rehabilitation actions:

(1) single-joint movement: choose ankle pump movement, ankle joint rotation movement of the 2 single-joint movement in the patient anesthetized awake immediately, both belong to the prevention of lower limb thrombosis rehabilitation actions easy to understand and the effect of a group of actions, the frequency of activities for 10 times/group, 10 groups/d.
(2) Simple muscle exercise: instruct patients to perform quadriceps muscle contraction exercise, to lay a good foundation for the later early movement out of bed, to avoid thigh muscle weakness, the frequency of activities for 10 times/group, 10 groups/d.
(3) Multi-joint movement: Patients mainly use the joint axis to pull the limb movement during joint movement, the joint chain refers to the composite chain composed of 2 or more parts of the human body connected through joints in a certain order.

The straight leg raising exercise after TKA belongs to the most common type of exercise in the open kinematic chain, which is characterized by exercising single or several joints at the same time, mainly showing that the proximal end of the limb is fixed, while the distal end is movable. One type of rehabilitation, the main feature of which is the immobilization of the knee and distal joints of the affected limb while the patient moves the proximal part of the limb, is more similar to functional rehabilitation. The combination of the 2 allows for a maximum range of motion (ROM) of the limb, providing early, effective and safe rehabilitation.

Paying attention to the rule of progression during rehabilitation exercises. The exercise follows the Tens rule, which consists of 10 seconds of contraction (the first 2 seconds to slowly increase the strength of the limb, the next 6 seconds to hold the contraction, and the last 2 seconds to gradually relax the limb), and 10 seconds in between to allow the patient to rest. 10 repetitions, 3 sets a day.^[12]^

## 3 Results

The kinesiophobia score and pain score of both the intervention group and the control group decreased as the length of hospitalization increased, and the decrease was more significant in the intervention group than in the control group. There was no difference in kinesiophobia and pain scores between the 2 groups on postoperative day 1 (*P* > .05), and there was a difference in kinesiophobia and pain scores between the 2 groups on postoperative days 3 and 7 (*P* < .01), and after repeated measures ANOVA, there was a difference in time point, group and interaction effect between the 2 groups (*P* < .01) (see Table 2).

**Table 2:** Comparison of cognitive indicators between 2 groups of patients.

Items	Groups	Day 1 after surgery	Day 3 after surgery	Day 7 after surgery	F_time_	F_group_	F_interactive_
TSK	Intervention	38.58 ± 1.74	36.00 ± 1.37	33.66 ± 1.59	208.400	10.907	14.815
	Control	38.61 ± 2.28	36.80 ± 1.73	35.75 ± 2.34			
	t	−0.058	−2.617	−5.238			
	P	0.954	< 0.01	<0.01	<0.01	<0.01	<0.01
Pain	Intervention	5.42 ± 1.05	2.91 ± 0.63	1.81 ± 0.74	380.976	31.438	5.016
	Control	5.61 ± 0.96	3.61 ± 0.85	2.75 ± 0.60			
	t	0.978	−4.802	−7.104			
	P	0.331	<0.01	<0.01	<0.01	<0.01	<0.01

TSK = Tampa Scale for kinesiophobia.

Patients should be accompanied by family members or medical personnel when they first get out of bed, and the walking distance after getting out of bed can be greater than or equal to 5 m with the help of a walking aid or family members, and the specific time started to be calculated immediately after the transfer from the anesthesia recovery room.^[10]^ The mean time to get out of bed for the first time in the intervention group (36.34 ± 13.42) was less than that in the control group (43.297 ± 18.45), and the difference was statistically significant (*P* < .05).

Joint mobility and daily living ability scale scores increased with the length of hospitalization in both groups, and the increase in the intervention group was more significant than that in the control group. There was no difference in ROM and activities of daily living scale (ADL) scores in the intervention group compared with the control group on postoperative day 1 (*P* > .05), and there was a difference on postoperative days 3 and 7 (*P* < .01), and a further repeated-measures ANOVA showed that there was a difference in time point, group, and interaction effect between joint mobility and the daily living ability scale (*P* < .01) (see Table 3).

**Table 3:** Comparison of behavioral indicators between 2 groups of patients.

Items	Groups	Day 1 after surgery	Day 3 after surgery	Day 7 after surgery	F_time_	F_group_	F_interactive_
ROM	Intervention	47.85 ± 15.75	68.13 ± 11.67	83.98 ± 8.23	349.846	7.184	5.099
	Control	46.01 ± 16.35	62.06 ± 11.37	74.41 ± 9.57			
	t	0.581	2.687	5.473			
	p	0.562	<0.01	<0.01	<0.01	<0.01	<0.01
ADL	Intervention	51.79 ± 7.28	79.06 ± 0.66	87.36 ± 4.34	979.755	28.162	5.086
	Control	49.90 ± 8.57	71.34 ± 7.93	82.69 ± 5.04			
	t	1.214	5.767	5.702			
	p	0.228	<0.01	<0.01	<0.01	<0.01	<0.01

ADL = activities of daily living scale.

## 4 Discussion

The current new coronary pneumonia epidemic is spreading around the world and has affected more than 200 countries and regions, and all countries are facing different degrees of epidemic prevention dilemmas, Internet technology played an important role during this fight against the new coronary pneumonia epidemic, and the effective use of the Internet has also become an indispensable tool in the work of medical personnel.^[13]^ WeChat, a tool used in this study to implement a micro-video intervention for patients, has the benefit that the availability of micro-video during an epidemic can effectively reduce or avoid direct contact between patients and health care workers, thus cutting off the transmission route.

Knee joint is the joint in the human body that carries a lot of weight and has a lot of movement. Studies show that about 90% of people over 50 years old have X-ray changes in the knee joint,^[14]^ and 75% of people over 65 years old have osteoarthritis.^[2]^ TKA has been widely accepted as an effective procedure for treating patients with end-stage osteoarthritis both at home and abroad. It is a procedure in which biological or non-biological materials are made into an artificial knee joint and inserted into the human body to treat end-stage knee disease.^[15]^ kinesiophobia^[16]^ is the fear that the body will be injured or aggravated by activities that cause the patient to be unusually sensitive or catastrophic to pain, which ultimately leads to an irrational, irrational, and even debilitating psychological state to movement or activity.^[17]^ The Dourypanchout study showed that kinesiophobia after knee arthroplasty is detrimental to the recovery of functional parameters such as maximum passive flexion mobility.^[18]^

Patients in the intervention group had lower pain scores and kinesiophobia scores than those in the control group on postoperative day 3 and 7 (*P* < .01), and the 2 groups differed in time point, group, and interaction by repeated measures ANOVA (*P* < .01). The results showed that the kinesiophobia score and pain score of the intervention group decreased with the increase of the length of hospital stay compared with the control group, indicating that micro-video intervention can reduce the fear psychology of the kinesiophobia patients after total knee arthroplasty during COVID-19, so as to relieve postoperative pain, which was consistent with Doury's results.^[19]^ The reason is that, compared with traditional health education, micro-video health education focuses on the interaction between patients’ acceptance and feedback in the communication process, which can reduce patients’ kinesiophobia by eliminating their negative beliefs; secondly, micro-videos are produced systematically throughout the patients’ hospitalization period to strengthen their self-rehabilitation and maintenance behaviors by closely integrating the knowledge of kinesiophobia and knee replacement. to motivate them to switch from passive exercise to voluntary training to avoid adverse consequences such as weakened knee function, joint adhesions, and lower extremity thrombosis.

The mean time to get out of bed for the first time was 36.34 hours in the intervention group, which was better than the 43.27 hours in the control group (*P* < .05), and the scores of joint mobility and self-care ability of daily life scale were higher in the intervention group than in the control group on postoperative day 3 and 7 (*P* < .01). The results showed that ROM and ADL of the intervention group and the control group increased significantly with the extension of hospital stay, indicating that micro-video intervention can promote the early and progressive functional exercise of the kinesiophobia patients after total knee arthroplasty during COVID-19, so as to promote rapid recovery. This result confirms that the rehabilitation exercise method provided by micro-video can enhance the strength of lower limb muscles of patients with kinesiophobia, which is conducive to timely independent walking. The results were consistent with Hande^[20]^ and Cai.^[21]^ With audio-rich and illustrated micro-videos for the intervention, the researchers’ timely follow-up during the patients’ exercise process helped to direct the patients’ attention from pain braking to active exercise, gradually transitioning from initial reluctance or fear of exercise to willingness to try until active rehabilitation exercise, increasing the patients’ participation in behavior management, visualizing the exercise outcomes, and gaining a sense of accomplishment in behavior management during the behavior remodeling process. Reach the set goal of postoperative knee function exercise as soon as possible to avoid poor knee function recovery in the patient.

## 5 Conclusion

The causes of postoperative pain after total knee arthroplasty are complex, and some studies have found that the arthroplasty surgery itself will cause patients to have strong psychological stress reactions, and these reactions and postoperative pain will affect each other, making the diagnosis and treatment of postoperative pain extremely difficult. Traditional rehabilitation instruction is usually nurse-led, but during neo-coronary pneumonia, the use of micro-video interventions can effectively avoid direct contact between patients and health care professionals. The research results showed that WeChat micro-video intervention could reduce the pain degree and kinesiophobia degree of patients with kinesiophobia after total knee arthroplasty during covid-19, and improve the ROM and the ADL. The shortcomings of this study were limited to observation during hospitalization, and there was a lack of mid- and long-term follow-up of patients after discharge from the hospital. In future studies, we may consider expanding the sample size, extending the time points of patient observation, and more scientifically evaluating the cognitive and behavioral adaptations of micro-communication on postoperative agoraphobic patients with TKA from a medical psychological perspective, so as to promote patients’ rapid recovery.

## Author contributions

All authors read and approved the final version of the manuscript.

**Conceptualization:** Guanzhen Lu, Tingting Wu, Qin tan, Yan Zhong.

**Investigation:** Zhe Wu, Lingmei Shi, Tingting Wu.

**Formal analysis:** Tingting Wu, Guanzhen Lu.

**Writing – original draft:** Guanzhen Lu, Tingting Wu.

**Writing – review & editing:** Yan Zhong.

## Abbreviations

Abbreviations: ADL = activities of daily living scale, ROM = range of motion, TKA = total knee arthroplasty.
